# Differential expression of miR-128 in memory T cells of patients with severe hemophilia A with and without inhibitors

**DOI:** 10.1016/j.rpth.2026.106639

**Published:** 2026-05-08

**Authors:** Silvia Spena, Andrea Cairo, Emanuela Pappalardo, Calogero C. Tedesco, Roberta Gualtierotti, Flora Peyvandi

**Affiliations:** 1 Fondazione IRCCS Ca’ Granda Ospedale Maggiore Policlinico, Angelo Bianchi Bonomi Hemophilia and Thrombosis Center, Milan, Italy; 2 Department of Pathophysiology and Transplantation, Università degli Studi di Milano, Milan, Italy

**Keywords:** hemophilia A, memory T cell, microRNAs, neutralizing antibodies, peripheral blood mononuclear cell

## Abstract

**Background:**

Inhibitor development occurs in ∼30% of persons with severe hemophilia A treated with factor (F)VIII replacement products, but the real mechanism of the development is poorly known. miR-128 is a crucial regulator of immune responses, and it is implicated in the pathobiology of various autoimmune disorders.

**Objectives:**

To investigate the expression of miR-128 in peripheral blood mononuclear cells (PBMCs) and CD4^+^ naïve and memory T cells of patients with severe hemophilia A with and without inhibitors.

**Methods:**

Forty-five patients with severe hemophilia A, >50 exposures to FVIII and with persistent (*n* = 9), transient (*n* = 10), or no inhibitor (*n* = 26) were enrolled. The whole blood was drawn to isolate PBMCs and then CD4^+^ naïve and memory T cells. Total RNA was extracted, and levels of miR-128 were evaluated in PBMCs, naïve T cells, and memory T cells by quantitative polymerase chain reaction. Analysis of covariance test was used to compare the expression of miR-128 in different inhibitor conditions.

**Results:**

A statistically significant increase in miR-128 levels in PBMCs (1.5-fold change; *P* = .027) and memory T cells (2.6-fold change; *P* = .015) was found in patients with (persistent and transient) inhibitors compared with patients without inhibitors. In particular, a higher expression of miR-128 in memory T cells (3.8-fold change; *P* = .022) was found in patients with a persistent inhibitor compared with patients without inhibitors.

**Conclusion:**

The present study indicates an overexpression of miR-128 in memory T cells of persons with severe hemophilia A developing inhibitors, likely regulating the expression of key genes in T cell differentiation.

## Essentials

• miR-128 regulates the immune response and is implicated in many autoimmune disorders.
• miR-128 expression was evaluated in persons with severe hemophilia A with and without inhibitors (INHs).
• miR-128 was increased in PBMCs and memory T cells of persons with (active and transient) INH.
• Among persons with INH, those with active INH had higher expression of miR-128 in memory T cells.

## 1 Introduction

Hemophilia A is a bleeding disorder characterized by plasma deficiency of clotting factor (F)VIII. Persons with severe hemophilia A and no measurable FVIII activity (<0.01 IU/mL) experience frequent and spontaneous bleeding episodes, mainly at joints and muscles [1,2]. Bleeding episodes are controlled through the infusion of plasma-derived or recombinant FVIII concentrates. Unfortunately, up to 30% of patients develop, mainly in the first 20 infusions, anti-FVIII neutralizing alloantibodies (ie, inhibitors [INHs]) that decrease the efficacy of the replacement therapy [3]. INH development follows stimulation of CD4^+^ T cells by exogenous FVIII, whereas follicular CD4^+^ T cells mediate maturation of B cells, class switching, and development of FVIII-specific IgG-secreting plasma cells and memory B cells [4]. The INH development has been described as a multifactorial complication resulting from environmental and genetic factors, among which null mutations in the FVIII coding gene, leading to the complete lack of FVIII activity, are to date the strongest risk factor [[5], [6], [7]]. Overall, the molecular basis of INH development remains poorly understood.

MicroRNA (miRNA) is a class of noncoding RNAs of 19 to 25 nucleotides that mainly control the posttranscriptional expression of protein-coding genes by interacting with the 3′ untranslated region of target mRNAs and by inducing mRNA degradation or translational repression [8]. The classical miRNA biogenesis pathway consists of transcription of intragenic/intergenic DNA sequence into a long primary miRNA containing multiple stem-loop structures, cleavage at the base of the primary miRNA hairpin by the DGCR8/Drosha complex to generate a precursor miRNA (pre-miRNA), export of the pre-miRNA to the cytoplasm and cleavage of the pre-miRNA loop by the endoribonuclease Dicer to produce mature miRNA duplex (ie, 5p and 3p strands). The strand with lower 5' stability is picked up by the AGO protein and the other strand undergoes degradation [9]. miR-128 is encoded by 2 intragenic sequences (miR-128-1 and miR-128-2) localized within introns of *R3HDM1* (2q21.3) and *ARPP-21* (3p22.3) genes, respectively. Although both pre-miR-128 form different stem-loop structures, after processing by Dicer they yield identical mature miR-128 (miR-128-3p) [10]. miR-128 is involved in several biological process including the immune response. Changes in the miR-128 expression and activity regulate immunologic responses and contribute to vulnerability to autoimmune disorders (ie, inflammatory bowel disease and rheumatoid arthritis) and neuroinflammatory disorders (Alzheimer disease, Parkinson disease, Huntington disease, epilepsy, and multiple sclerosis) [11]. Increased levels of miR-128 have been described in T cells of patients with rheumatoid arthritis [12], monocytes and lymphocytes of patients with Alzheimer disease [13], and in naïve CD4^+^ T cells of patients with multiple sclerosis [14]. In this study, the expression of miR-128 was evaluated in PBMCs, CD4^+^ naïve T cells, and CD4^+^ memory T cells of 45 patients with severe hemophilia A with and without INH.

## 2 Methods

### 2.1 Patients

From March 2021 to June 2023, 45 unrelated male patients with severe hemophilia A (FVIII:C <1%), regularly followed at the Angelo Bianchi Bonomi Hemophilia and Thrombosis Center, Fondazione Istituto di Ricovero e Cura a Carattere Scientifico (IRCCS) Ca’ Granda, Ospedale Maggiore Policlinico (Milan, Italy), were consequently enrolled. All patients had received at least 50 infusions of FVIII during on-demand or prophylactic treatment. At the enrollment, patients were not undergoing immune tolerance induction therapy and did not experience inflammatory disorders, coronary artery disease, or cancer. Twenty-six patients that never developed INH were classified as INH negative (INH^−^); 19 patients that before or at the enrollment time had a measurable INH (>0.5 Bethesda Units/mL [15]) were classified as INH positive (INH^+^). Among the INH^+^ patients, 10 had developed a transient (ie, temporary) INH and were subclassified as INHt^+^; 9 had an active (ie, current) INH and were subclassified as INHa^+^. Demographic and clinical characteristics of enrolled patients are reported in the Table.

**Table:** Demographic and clinical characteristics of enrolled patients.

ID	INH status	INH titer^a^	ITI^b^	Age (y)^c^	Country
1	INHt^+^	1	Effective	28	Italy
2	INHt^+^	1	No	25	Italy
3	INH^−^	−	−	49	Italy
4	INH^−^	−	−	45	Italy
5	INH^−^	−	−	28	Italy
6	INH^−^	−	−	58	Italy
7	INHt^+^	1	Effective	70	Italy
8	INH^−^	−	−	56	Italy
9	INH^−^	−	−	23	Italy
10	INH^−^	−	−	74	Italy
11	INH^−^	−	−	37	Italy
12	INHt^+^	0.9	Effective	67	Italy
13	INH^−^	−	−	19	Italy
14	INH^−^	−	−	36	Italy
15	INHt^+^	2	Failed	27	Italy
16	INH^−^	−	−	57	Italy
17	INHa^+^	12	Failed	49	Italy
18	INHa^+^	1	Failed	50	Italy
19	INHa^+^	2	Failed	23	Egypt
20	INH^−^	−	−	35	Argentina
21	INH^−^	−	−	39	Italy
22	INH^−^	−	−	42	Italy
23	INH^−^	−	−	23	Italy
24	INHa^+^	6	Failed	8	China
25	INHa^+^	12.5	No	12	Senegal
26	INHa^+^	1	No	14	Italy
27	INHa^+^	4	Failed	43	Italy
28	INHt^+^	2	Effective	16	Italy
29	INHt^+^	2	Effective	45	Italy
30	INHt^+^	1	Effective	22	Philippines
31	INHt^+^	3	No	34	Egypt
32	INHt^+^	11	Effective	28	Italy
34	INH^−^	−	−	44	Italy
35	INH^−^	−	−	32	Italy
36	INH^−^	−	−	50	Italy
37	INH^−^	−	−	38	Italy
38	INH^−^	−	−	50	Italy
40	INH^−^	−	−	42	Italy
41	INHa^+^	9.5	No	63	Italy
42	INH^−^	−	−	45	Italy
43	INH^−^	−	−	25	Italy
44	INH^−^	−	−	49	Italy
45	INH^−^	−	−	48	Italy
46	INHa^+^	6	Failed	22	Ecuador
47	INH^−^	−	−	41	Italy

ID, patient identification number; INH, inhibitor; ITI, immune tolerance induction. INH^−^, INHa^+^, and INHt^+^ denote an INH never developed, developed and persistent, and developed in the past and no longer measurable, respectively; − denotes a not applicable information.

a Current and last positive inhibitor in INHa^+^ and INHt^+^ patients, respectively, are reported in Bethesda Units/mL.

b ITI outcome (effective/failed) and absence of ITI therapy (no) are reported.

c Age at blood sampling.

Written informed consent was obtained for all patients. The study was conducted in accordance with the Declaration of Helsinki and approved by the Institutional Review Board of the Fondazione IRCCS Ca’ Granda, Ospedale Maggiore Policlinico, Milan, Italy (206_2018bis; April 6, 2018).

### 2.2 Isolation of PBMCs, naïve T cells, and memory T cells

Peripheral blood (32 mL) was drawn by clean venipuncture of the antecubital veins in Vacutainer CPT tubes (BD Biosciences). After centrifugation at 1700 *g* for 30 minutes at room temperature, PBMCs were collected and washed 3 times with phosphate buffer saline; one-eighth of PBMCs were suspended in 500 μL of Qiazol Lysis Solution (Qiagen) and stored at −80 °C until the RNA extraction. The remaining PBMCs were equally distributed to undergo the isolation of naïve and memory T cells by means of the human naïve CD4^+^ T Cell Isolation Kit II and the Memory CD4^+^ T cell isolation kit (Miltenyi), respectively, according to the manufacturer’s instructions. Double staining with antihuman CD4-APC/CD45RA-FITC and CD4-APC/CD45RO-FITC antibodies (Miltenyi) were performed to evaluate by flow cytometry the purity of naïve and memory T cells, respectively.

### 2.3 RNA extraction and expression analysis of miR-128

Total RNA was isolated from PBMCs, naïve and memory T cells using the miRNeasy kit (Qiagen). The miR-128-3p, miR-128-5p, and the RNU6B were reverse transcribed with their respective miRNA-specific primers and the TaqMan MicroRNA Reverse Transcription Kit (Thermo Fisher). Expression analysis of the miR-128-3p, miR-128-5p, and the housekeeping RNU6B was performed on 13 ng of miRNA-specific cDNA using Taq-Man assays 002216, 466976, and 001093, respectively, and TaqMan Fast Advanced Master Mix (Thermo Fisher). Fluorescence signals were monitored using the StepOnePlus Real-Time PCR system (Applied Biosystems) and StepOne v2.3 software (Applied Biosystems). Two duplicates of each sample were analyzed, and the relative quantities were determined using the 2^−ΔΔCt^ method.

### 2.4 Statistical analysis

Analysis of covariance (ANCOVA) was used to compare miRNA expression across groups (INH^+^ vs INH^−^ and INHa^+^ vs INHt^+^ vs INH^−^) taking into account for covariates variability with Tukey-Kramer post hoc test to perform pairwise comparisons between all groups.

## 3 Results and Discussion

Many studies have been performed trying to identify genetic and environmental factors predisposing to INH development in hemophilia, but the majority of them, such as duration and dose of treatment at initial exposure, type of replacement product, and polymorphisms in immune response genes, have been described with conflicting results [16]. Despite null mutations in the *F8* gene and a positive family history are ascertained as major risk factors, the complication remains largely unpredictable and the mechanism of development currently poorly known. miRNAs regulate posttranscriptional gene expression in many cells including those of the immune system, thus managing the proliferation and differentiation of B cells and the development, repair, and activation of T cells [17]. However, their role on INH development has been poorly investigated in persons with hemophilia. To the best of our knowledge, only 1 study addressed this point by performing ncRNA microarrays and quantitative polymerase chain reaction (qPCR) analysis on RNA extracted from whole blood samples of a small number of patients with hemophilia A (3 with and 6 without INHs) [18]. The authors reported a 6-fold lower expression of miR-1246 in patients with INHs than in patients without INHs. Since miR-1246 is known to be downregulated in human natural killer cells stimulated with interleukin (IL) 2, IL-15, and IL-21 [19], and it directly targets the *F8* transcript [18], the authors suggested a possible role of miR-1246 in hemophilia pathophysiology and in variable responses to FVIII-replacement therapy. To improve knowledge about the possible role of miRNAs in INH development, we focused on miR-128, whose overexpression or lowerexpression has been associated with many neuroinflammatory and autoimmune disorders [11]. To this aim, we specifically studied the expression of miR-128 in immune cells: PBMCs, mainly consisting in lymphocytes; and the lymphocyte subpopulations of naïve and memory CD4^+^ T cells where miR-128 was previously found dysregulated in patients with multiple sclerosis [14].

We analyzed cells of 45 persons with severe hemophilia A that, after >50 exposures to FVIII, showed an active (INHa^+^; 9), a past (INHt^+^; 10), or a never developed (INH^−^; 26) INH (Table). At the time of blood collection, all patients did not have other comorbidities that are known to alter levels of miRNAs. The median age of available patients was slightly higher in the INH^−^ group than in the INHa^+^ and INHt^+^ groups (41.7 vs 31.6 and 36.2 years, respectively) (Table). Since the expression of miR-128 in PBMCs was reported to decrease with age [20], analysis of miR-128 expression was adjusted for age. The majority (84.4%) of enrolled patients were Italian (Table). As expected, the only 3 persons with an ethnicity (Black or Hispanic) known to have a higher risk of INH development [21] were positive for INH (Table). Since no correlation between ethnicity of patients and miR-128 levels was found, no further adjustment was needed.

All RNA samples extracted from PBMCs were suitable for the qPCR amplification; only 1 (patient 25; INHa^+^) failed the amplification and was excluded from the analysis (Table). Despite the high starting volume of blood, very few RNA was extracted from naïve and memory T cells of 9 patients (patient 46, INHa^+^; patient 12, INHt^+^; and patients 10, 14, 37, 42-45, INH^−^) (Table). Therefore, qPCR amplification and analysis were finally performed on PBMCs of 44 patients (18 with and 26 without INH) and on naïve and memory T cells of 36 patients (8 with persistent, 9 with transient, and 19 without INH).

**Figure 1:**
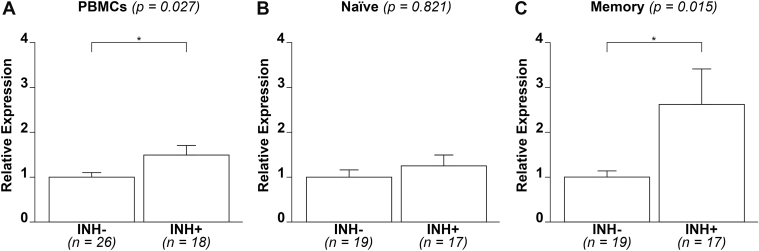
Box plots show the relative expression (fold change) of miR-128-3p in PBMCs (A), CD4^+^ naïve (B), and memory (C) T cells of patients with severe hemophilia A with (INH^+^) and without (INH^−^) inhibitor. The *P* value and the number (n) of analyzed individuals for each inhibitor condition are between parentheses: ∗*P* < .05.

miR-128-5p was not detected since probably degraded in the analyzed cells. By contrast, a statistically significant increase in miR-128 levels was found in PBMCs (1.5-fold change; *P* = .027) and, to a greater extent, in the subpopulation of memory T cells (2.6-fold change; *P* = .015) of patients with INH (INH^+^) compared with patients without INH (INH^−^) (Figure 1A, C). No difference in miR-128-3p expression was found in naïve T cells of patients with (INH^+^) and without (INH^−^) INH (Figure 1B). Since memory T cells are the most abundant cell type in PBMCs (up to 30%), the small increase observed in these cells could be driven by memory T cells. Moreover, the fact that miR-128 was quantitatively modified in CD4^+^ memory T cells of patients with hemophilia who have developed INH but not in CD4^+^ naïve (i.e. never activated) T cells may suggest that the high expression of miR-128 is not genetically determined but most likely involved in the molecular pathway of INH development. This is also supported by a recent report of miRNA core signatures of CD4^+^ memory and naïve T cells from healthy subjects [22], which does not list miR-128.

**Figure 2:**
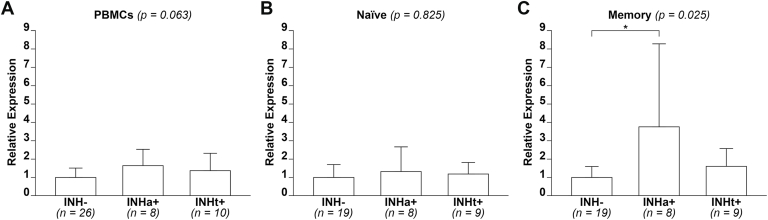
Box plots show the relative expression (fold change) of miR-128-3p in PBMCs (A), CD4^+^ naïve (B) and memory (C) T cells of patients with severe hemophilia A with active (INHa^+^), transient (INHt^+^), and without (INH^−^) inhibitor. The analysis of covariance *P* and the number (*n*) of analyzed individuals for each inhibitor condition are between parentheses: ∗*P* < .05.

To evaluate possible difference in miR-128 expression in the INHa^+^ and INHt^+^ subconditions, a multiple (INH^−^, INHa^+^, and INHt^+^) comparison was performed in PBMCs, naïve T cells, and memory T cells (Figure 2). The analysis was statistically significant only in memory T cells (ANCOVA, *P* = .025) (Figure 2C). Pairwise comparison confirmed the overexpression of miR-128 in memory T cells (3.8-fold change; *P* = .022) of patients with active INH (INHa^+^) compared with patients without INH (INH^−^) (Figure 2C), while no statistically significant differences were found in memory T cells of patients with a past INH (INHt^+^) compared with those with active (INHa^+^) or without (INH^−^) INH (Figure 2C). Since miR-128 levels in INHt^+^ patients seem intermediate compared with levels in INHa^+^ and INH^−^ patients (Figure 2), it could be speculated that the immunologic response (ie, the INH production) might occur once a threshold value of miR-128 is exceeded. While the upregulation observed in CD4^+^ memory T cells of INHa^+^ vs INH^−^ patients with hemophilia A is supported by a statistical power >80%, the small and not statistically significant differences observed in memory T cells of INHt^+^ patients vs INHa^+^ and vs INH^−^ patients are not. A larger sample size would allow to better understand whether the miR-128 signature in memory T cells of patients with hemophilia A and a past INH (INHt^+^) resemble to the INHa^+^ or to the INH^−^ condition. Hence, the main limitation of the study is the relative low number of analyzed patients. It is caused by the overall low incidence of patients (i) with severe hemophilia A (1-9 in 100,000; Orphanet; https://www.orpha.net/en/disease/detail/169802); (ii) who develop the INH (∼30%); (iii) who spontaneously or after immune tolerance induction resolve it and, even more, by the reduced recovery efficiency (∼80%) of RNA obtained from purified naïve and memory CD4^+^ T cells that probably relies on the intrinsic low and variable frequency of these cells in PBMCs.

To our knowledge, this study is the first to report an overexpression of miR-128 in highly specific CD4^+^ memory T cells. A high expression of miR-128 has been previously found in other immunologic cells, such as naïve CD4^+^ T cells of patients with multiple sclerosis [14], monocytes and lymphocytes of patients with Alzheimer disease [13], and T cells of patients with rheumatoid arthritis [12]. Moreover, the role of miR-128 in the inflammation response has been previously investigated. In multiple sclerosis, miR-128 was reported to inhibit Th2 cell development and favor proinflammatory Th1 responses by directly suppressing BMI1 (B lymphoma Mo-MLV insertion region 1 homolog) [14], which stabilizes the Th2 transcription factor GATA binding protein 3 in T cells [23]. In rheumatoid arthritis, downregulation of miR-128 has been demonstrated to suppress the nuclear factor κB pathway and the inflammation response through TNFAIP3 (tumor necrosis factor, α-induced protein 3), which is a direct target of miR-128 [12]. Moreover, since monocytes are potentially the antigen presenting cells in FVIII immune responses, it would be interesting to evaluate miR-128 levels also in this cell type.

In conclusion, despite the aforementioned limitations, this study provides evidence of overexpression of miR-128 in CD4^+^ memory T cells of persons with severe hemophilia A with an active INH and pave the way for future investigations to confirm and understand the effective contribution of miR-128 in INH development and memory cell maintenance in patients with severe hemophilia A.

## References

[1] Mannucci P.M., Tuddenham E.G. (2001). The hemophilias—from royal genes to gene therapy. N Engl J Med.

[2] Peyvandi F., Garagiola I., Young G. (2016). The past and future of haemophilia: diagnosis, treatments, and its complications. Lancet.

[3] Witmer C., Young G. (2013). Factor VIII inhibitors in hemophilia A: rationale and latest evidence. Ther Adv Hematol.

[4] Jing W., Chen J., Cai Y., Chen Y., Schroeder J.A., Johnson B.D. (2019). Induction of activated T follicular helper cells is critical for anti-FVIII inhibitor development in hemophilia A mice. Blood Adv.

[5] Spena S., Garagiola I., Cannavò A., Mortarino M., Mannucci P.M., Rosendaal F.R. (2018). Prediction of factor VIII inhibitor development in the SIPPET cohort by mutational analysis and factor VIII antigen measurement. J Thromb Haemost.

[6] Oldenburg J., Pavlova A. (2006). Genetic risk factors for inhibitors to factors VIII and IX. Haemophilia.

[7] Gouw S.C., van den Berg H.M., Oldenburg J., Astermark J., de Groot P.G., Margaglione M. (2012). F8 gene mutation type and inhibitor development in patients with severe hemophilia A: systematic review and meta-analysis. Blood.

[8] Ying S.Y., Chang D.C., Lin S.L. (2008). The microRNA (miRNA): overview of the RNA genes that modulate gene function. Mol Biotechnol.

[9] O’Brien J., Hayder H., Zayed Y., Peng C. (2018). Overview of microRNA biogenesis, mechanisms of actions, and circulation. Front Endocrinol (Lausanne).

[10] Kiel K., Król S.K., Bronisz A., Godlewski J. (2024). MiR-128-3p—a gray eminence of the human central nervous system. Mol Ther Nucleic Acids.

[11] Margiana R., Kzar H.H., Hussam F., Hameed N.M., Al-Qaim Z.H., Al-Gazally M.E. (2023). Exploring the impact of miR-128 in inflammatory diseases: a comprehensive study on autoimmune diseases. Pathol Res Pract.

[12] Xia Z., Meng F., Liu Y., Fang Y., Wu X., Zhang C. (2018). Decreased MiR-128-3p alleviates the progression of rheumatoid arthritis by up-regulating the expression of TNFAIP3. Biosci Rep.

[13] Tiribuzi R., Crispoltoni L., Porcellati S., Di Lullo M., Florenzano F., Pirro M. (2014). miR128 up-regulation correlates with impaired amyloid β(1-42) degradation in monocytes from patients with sporadic Alzheimer’s disease. Neurobiol Aging.

[14] Guerau-de-Arellano M., Smith K.M., Godlewski J., Liu Y., Winger R., Lawler S.E. (2011). Micro-RNA dysregulation in multiple sclerosis favours pro-inflammatory T-cell-mediated autoimmunity. Brain.

[15] Verbruggen B., van Heerde W., Novákovà I., Lillicrap D., Giles A. (2002). A 4% solution of bovine serum albumin can be used in place of factor VIII:C deficient plasma in the control sample in the Nijmegen Modification of the Bethesda factor VIII:C inhibitor assay. Thromb Haemost.

[16] Garagiola I., Palla R., Peyvandi F. (2018). Risk factors for inhibitor development in severe hemophilia A. Thromb Res.

[17] Baumjohann D., Ansel K.M. (2013). MicroRNA-mediated regulation of T helper cell differentiation and plasticity. Nat Rev Immunol.

[18] Sarachana T., Dahiya N., Simhadri V.L., Pandey G.S., Saini S., Guelcher C. (2015). Small ncRNA expression-profiling of blood from hemophilia A patients identifies miR-1246 as a potential regulator of factor 8 gene. PLoS One.

[19] Liu X., Wang Y., Sun Q., Yan J., Huang J., Zhu S. (2012). Identification of microRNA transcriptome involved in human natural killer cell activation. Immunol Lett.

[20] Noren Hooten N., Abdelmohsen K., Gorospe M., Ejiogu N., Zonderman A.B., Evans M.K. (2010). microRNA expression patterns reveal differential expression of target genes with age. PLoS One.

[21] Ahmed A.E., Pratt K.P. (2023). Race, ethnicity, F8 variants, and inhibitor risk: analysis of the “My Life Our Future” hemophilia A database. J Thromb Haemost.

[22] Culina S., Commère P.H., Turc E., Jouy A., Pellegrini S., Roux T. (2025). MicroRNA signatures of CD4+ T cell subsets in healthy and multiple sclerosis subjects determined by small RNA-sequencing. J Neuroimmunol.

[23] Hosokawa H., Kimura M.Y., Shinnakasu R., Suzuki A., Miki T., Koseki H. (2006). Regulation of Th2 cell development by Polycomb group gene bmi-1 through the stabilization of GATA3. J Immunol.

